# HIV vulnerabilities and psychosocial health among young transgender women in Lima, Peru: results from a bio‐behavioural survey

**DOI:** 10.1002/jia2.26299

**Published:** 2024-07-23

**Authors:** Alfonso Silva‐Santisteban, Dorothy Apedaile, Amaya Perez‐Brumer, Segundo R. Leon, Leyla Huerta, Francezka Leon, Rodrigo Aguayo‐Romero, Sari L. Reisner

**Affiliations:** ^1^ Center for Interdisciplinary Research in Sexuality AIDS and Society Universidad Peruana Cayetano Heredia Lima Peru; ^2^ Dalla Lana School of Public Health University of Toronto Toronto Ontario Canada; ^3^ Escuela Profesional de Tecnología Médica Universidad Privada San Juan Bautista Lima Peru; ^4^ Feminas Peru Lima Peru; ^5^ Division of Endocrinology Diabetes, and Hypertension Brigham and Women's Hospital Boston Massachusetts USA; ^6^ Department of Medicine Harvard Medical School Boston Massachusetts USA; ^7^ Department of Epidemiology Harvard T.H. Chan School of Public Health Boston Massachusetts USA; ^8^ The Fenway Institute Fenway Health Boston Massachusetts USA

**Keywords:** transgender women, youth, HIV, mental health, epidemiology, vulnerability

## Abstract

**Introduction:**

Peruvian young transgender women (YTW) ages 16−24 years are a critical but understudied group for primary HIV prevention efforts, due to sharp increases in HIV prevalence among TW ages 25 years and older.

**Methods:**

Between February and July 2022, a cross‐sectional quantitative study with YTW ages 16−24 years in Peru (*N* = 211) was conducted consisting of a bio‐behavioural survey accompanied by laboratory‐based testing for HIV and sexually transmitted infections (STIs). Bivariate and multivariable Poisson regression models were used to estimate prevalence ratios between socio‐demographic and behavioural characteristics and HIV status.

**Results:**

HIV prevalence was 41.5% (95% CI: 33.9−49.4%), recent syphilis acquisition 19.4% (95% CI: 12.7−28.4), chlamydia 6.3% (95% CI: 3.1−11.1) and gonorrhoea 12.3% (95% CI: 7.9−18.7). Almost half (47.9%) reported condomless anal sex in the past 6 months, 50.7% reported sex work in the past 30 days and 13.7% reported accepting more money for condomless sex. There were no significant differences in reported sexual behaviours by HIV status. Only 60.8% of participants reported ever having been tested for HIV, and 25.6% reported a past 6‐month STI test. More than two‐thirds (67.8%) had not heard of antiretroviral pre‐exposure prophylaxis (PrEP) and only 4.7% had taken PrEP in the past month. Current moderate‐to‐severe psychological distress was endorsed by 20.3%, 10.0% reported attempting suicide in the past 6 months and 85.4% reported alcohol misuse.

**Conclusions:**

Findings show that the HIV epidemic for YTW in Lima, Peru is situated in the context of widespread social exclusion, including economic vulnerabilities, violence victimization and the mental health sequelae of transphobic stigma that starts early in life. Future research should aim to further understand the intersection of these vulnerabilities. Moreover, there is an urgent necessity to design and evaluate HIV prevention programmes that address the root systems driving HIV vulnerabilities in YTW and that focus on developmentally specific clusters of stigma‐related conditions.

## 1 INTRODUCTION

Globally, transgender women (TW) are at high risk for HIV acquisition with an estimated HIV prevalence, through a meta‐analysis combining available international data, of 19.1%, a nearly 50‐fold increased odds of HIV compared to the general population [1]. In Peru, TW are most affected by HIV, with a prevalence of 20.8−29.8%, measured in Lima and other cities of the country between 2009 and 2016 [2, 3], compared with 0.2−0.3% in the general population [3]. Young TW (YTW) ages 16−24 years are a critical group for primary HIV prevention efforts in Peru, due to sharp increases in HIV prevalence among TW ages 25 years and older, increasing four times the odds of HIV acquisition compared to the group 18−20 [2].

Biological risk factors associated with HIV transmission in TW include sexually transmitted infections (STIs) and condomless receptive anal sex [4, 5]. However, data are needed on HIV co‐occurrence with other STIs, and HIV risks specifically among YTW in Peru. Additionally, HIV risk for TW occurs in the context of widespread stigma and social exclusion, such as employment discrimination often associated with engagement in sex work, and internalization of transphobic mistreatment [6, 7]. Consequently, HIV acquisition is only one of the multiple stigma‐related health conditions that TW face [9]. The extent to which YTW in Peru are burdened by stigma, mental health (e.g. psychological distress, post‐traumatic stress disorder) and other psychosocial risks (e.g. gender‐based violence, alcohol misuse) remains largely unknown [9, 10].

Vulnerability for HIV in YTW occurs during the crucial developmental milieu of adolescence and young adulthood [11]. YTW navigate developmental tasks common to all youth (e.g. autonomy) [12]. They must also contend with tasks specific to being a transgender youth that may increase HIV risk [13] such as transgender identity disclosure (e.g. family, peers, romantic relationships), youth‐specific stigma exposures (e.g. transphobic bullying), and lack of youth‐ and trans‐friendly healthcare (e.g. HIV/STI biomedical prevention testing and treatment). Mental health and psychosocial vulnerabilities may co‐occur and synergistically potentiate risk for HIV in YTW [14] influencing bio‐behavioural HIV prevention goals such as protected sex and pre‐exposure prophylaxis (PrEP) indication [9]. Consequently, it is critical to characterize these vulnerabilities to design developmentally specific stigma‐related programmes that effectively mitigate HIV risks. Nonetheless, data regarding YTW and the HIV epidemic are still scarce in Peru and in Latin America [15].

Reducing HIV inequities for TW globally will require early HIV prevention efforts focusing on TW developmental risks uniquely occurring during adolescence and young adulthood. This paper characterizes the prevalence of HIV and other STIs, identifies structural and psychosocial vulnerabilities for HIV, and estimates the correlates of HIV status among YTW in Lima, Peru.

## 2 METHODS

### 2.1 Participants

Between February and July 2022, a cross‐sectional quantitative study was conducted with 211 YTW ages 16−24 in Peru consisting of a socio‐demographic, psychosocial and sexual risk behaviour survey accompanied by laboratory‐based testing for HIV, syphilis, gonorrhoea, chlamydia, hepatitis B and C. Eligibility criteria included identifying as a transgender woman, being age 16−24 years and residing in Lima. Being a transgender woman was defined as a person assigned a male sex at birth who identifies on the transfeminine continuum regardless of the initiation or completion of any medical gender affirmation procedures.

### 2.2 Community engagement

This study was informed by a formative qualitative research phase described elsewhere [16]. The qualitative phase of the study informed aspects of the study such as operating hours, incentives and duration of the survey. In addition, the study protocol was presented to Feminas, a community‐based organization formed and led by TW in Lima, to elicit community feedback on its feasibility and acceptability.

### 2.3 Recruitment and study sites

Participants were recruited via leaders or recruiters from a community‐based organization, and through peers. Participants who completed the study were also asked to refer potential participants to their social network to enhance the diversity of participants and expand recruitment. Six study offices were placed in six different districts giving the study wide coverage of metropolitan Lima. A fieldwork team was formed consisting of two survey interviewers, a laboratory technician and one counsellor. Survey interviewers were TW in their early 20s.

### 2.4 Measures

Survey measures were drawn from prior research with TW populations [17, 18, 19]. The interviewer‐administered survey explored socio‐demographic characteristics including age, educational level, region of birth, ethnic ancestry, work and employment, and monthly income. Medical gender affirmation was asked including hormone use, surgical procedures and industrial silicone injection (“silicone fillers”) for feminization. The questionnaire explored sexual behaviours such as sexual role (insertive, receptive, both), number of partners in the last 6 months, condomless sex, engagement in sex work and acceptance of money for not using condoms in sex work using measures from prior research with TW. Experiences of violence were assessed by asking about physical, psychological or sexual violence (ever and in the past 3 months) and transgender‐specific intimate partner violence, conceptualized as transphobic violence perpetrated by an intimate partner (e.g. “Did your partner tell you or threaten to tell someone else that you are transgender against your will, in order to humiliate you or to make you feel unsafe?”) [20]. The questionnaire also explored psychological distress using the six‐item Kessler‐6 psychological distress tool [21], post‐traumatic stress disorder using the five‐item Primary Care Post‐Traumatic Stress Disorder (PTSD) Screen for DSM‐V post‐traumatic stress disorder symptoms, suicidal thoughts and attempts [22], and alcohol misuse (using the AUDIT‐C score) [23]. Healthcare access was measured by assessing if the person had medical insurance, access to HIV/STI testing, PrEP awareness and use, and anticipated discrimination using the Intersectional Discrimination Index [24]. The final survey instrument was designed to take approximately 60 minutes to complete.

### 2.5 Procedures

Study staff explained the study process to eligible participants and obtained written consent before the face‐to‐face administration of the survey. Upon survey completion, trained personnel offered participants free voluntary counselling and testing for HIV, syphilis, chlamydia, gonorrhoea, hepatitis B and C. A blood sample consisting of 10 ml was collected by a trained lab technologist and stored for subsequent analysis. Results and post‐test counselling were delivered at study sites for (HIV and syphilis) or via text messages 1 week after specimen collection (for chlamydia, gonorrhoea, hepatitis B and C). Individuals testing positive for HIV were assisted with enrolling in the National HIV Program where HIV care and treatment, including antiretrovirals, are provided free of charge. Individuals testing positive for syphilis, chlamydia and gonorrhoea were referred to the public health clinic in their home jurisdiction to receive treatment. Participants who completed the survey received S/. 50 (approximately $13 USD) and a box of 100 condoms. Participants who referred other individuals received S/. 15 (approximately $4 USD) per participant referred up to three.

### 2.6 Laboratory procedures

HIV testing was performed by using two rapid HIV tests ([*Alere Determine™ HIV‐1/2 Ag/Ab Combo*—Alere, Waltham, MA, USA] and *SURE CHECK® HIV 1/2 Assay* [Chembio Diagnostic Systems Inc, NY, USA]) in parallel. Pre‐test counselling was provided by a certified HIV test counsellor following Peruvian guidelines. Confirmatory testing was performed via a combination of regular enzyme immunoassay and Western blot (Genscreen ULTRA HIV Ag‐Ab Assay and NEW LAB‐BLOT HIV‐1, BioRad, France). Those diagnosed as HIV positive were referred to the National Antiretroviral Treatment (Programa TARGA). Syphilis testing included qualitative and quantitative Rapid Plasma Reagin (RPR) tests followed by confirmation through Treponema pallidum‐Particle Agglutination test (TPPA) using a cut‐off value of 1:80. Hepatitis B and C virus testing were carried out using lateral flow rapid tests for HCV (SD Bioline, ABBOT Diagnostics) and DETERMINE™ HBsAg 2 (ALERE, ABBOT Diagnostics).

### 2.7 Data analysis

Proportions and 95% confidence intervals (95% CI) were estimated for binary and categorical variables of interest, while medians and interquartile ratios were estimated for continuous variables. Demographic, behavioural and biological outcome variables were analysed in relation to HIV status as the outcome of interest. Results are presented for the total study population that completed the survey as well as among only those participants who completed HIV testing. Bivariate analysis was conducted using chi‐squared tests for all variables except when the observed count was less than five, in which case a Fisher's exact test was used. The Cochran‐Armitage test was used to test for a trend in HIV prevalence by age group. Missing data were included as an explicit category in categorical bivariate comparisons and excluded from continuous bivariate comparisons. For the multivariable analysis, participants missing data on one or more variables of interest were excluded. Bivariate and multivariable Poisson regression models were used to estimate prevalence ratios representing the association between socio‐demographic and behavioural characteristics and HIV status. Robust standard errors were used to estimate 95% confidence intervals. The multivariable logistic regression model of HIV acquisition (positive/negative) included variables from the bivariate analysis which had a moderate association (*p*<0.2) with HIV acquisition. All analyses were conducted using R version 4.3.0. The full study protocol was approved by the Universidad Peruana Cayetano Heredia (Protocol #20606: 359‐35).

## 3 RESULTS

### 3.1 Sample characteristics

Out of 218 potential participants screened, 211 TW enrolled in the study and 164 (77.7%) gave blood samples for HIV/STI testing. There were no significant differences between those who completed HIV/STI testing and those who did not regarding socio‐demographic characteristics, self‐reported HIV status and previous HIV testing (Table S1). However, participants who gave a blood sample were significantly more likely to report engaging in recent condomless sexual activity (*p* = 0.02) and having multiple sexual partners (*p* = 0.02) (Table S1).

**Table 1:** Characteristics of young transgender women in Lima, Peru, overall and by HIV status

	All participants	Participants who completed HIV testing		
	Overall	HIV negative	HIV positive	
	*N* = 211	*N* = 96	*N* = 68	
	*n* (%)	*n* (%)	*n* (%)	*p*‐value^+^
Age in years (median [IQR])^a^	23.0 (21.0−24.0)	23.0 (20.0–24.0)	23.0 (21.0−24.0)	0.2
Born in Lima/Callao				0.87
Yes	94 (44.5%)	42 (43.8%)	27 (39.7%)	
No	110 (52.1%)	51 (53.1%)	39 (57.4%)	
Missing	7 (3.3%)	3 (3.1%)	2 (2.9%)	
Highest level of education				0.35
Elementary school or less	11 (5.2%)	3 (3.1%)	5 (7.4%)	
Secondary school, incomplete	47 (22.3%)	23 (24.0%)	15 (22.1%)	
Secondary school, complete	76 (36.0%)	32 (33.3%)	29 (42.6%)	
Post‐secondary, incomplete	58 (27.5%)	29 (30.2%)	14 (20.6%)	
Post‐secondary, complete	18 (8.5%)	9 (9.4%)	4 (5.9%)	
Missing	1 (0.5%)	0 (0.0%)	1 (1.5%)	
Currently studying	39 (18.57%)	19 (19.79%)	7 (10.45%)	0.13
Ancestry and customs				0.34
Indigenous Andean	7 (3.3%)	3 (3.1%)	2 (2.9%)	
Indigenous Amazonian	57 (27.0%)	22 (22.9%)	22 (32.4%)	
Indigenous (other)	10 (4.7%)	3 (3.1%)	4 (5.9%)	
Afroperuvian	26 (12.3%)	17 (17.7%)	6 (8.8%)	
White	15 (7.1%)	9 (9.4%)	2 (2.9%)	
Mestiza	72 (34.1%)	32 (33.3%)	23 (33.8%)	
Other	3 (1.4%)	2 (2.1%)	1 (1.5%)	
Missing	21 (10.0%)	8 (8.3%)	8 (11.8%)	
Employment status				0.82
Full time	15 (7.1%)	8 (8.3%)	4 (5.9%)	
Part time	9 (4.3%)	4 (4.2%)	2 (2.9%)	
Informal	112 (63.1%)	49 (51.0%)	29 (57.4%)	
Unemployed	71 (33.6%)	33 (34.4%)	21 (30.9%)	
Missing	4 (1.9%)	2 (2.1%)	2 (2.9%)	
Monthly household income				0.08
<300 soles	43 (20.4%)	28 (29.2%)	9 (13.2%)	
300–500 soles	51 (24.2%)	22 (22.9%)	17 (25.0%)	
501–1500 soles	50 (23.7%)	19 (19.8%)	20 (29.45)	
>1501 soles	31 (14.7%)	16 (16.7%)	7 (10.3%)	
Missing	36 (17.1%)	11 (11.5%)	15 (22.1%)	

Abbreviation: IQR, interquartile range.

^a^ One participant was missing age data.

^+^ *p*‐values of the comparison of proportions between participants who tested HIV positive and HIV negative.

Table 1 presents socio‐demographic characteristics overall and by HIV status among participants who completed HIV/STI testing. The median age of participants was 23 years (minimum 16, maximum 24). Approximately half of the participants (52.1%) were born outside of Lima, 72.0% of the participants had completed secondary school or higher and 18% reported currently being in school. Regarding ancestry, 34.1% of participants self‐identified as mixed (*mestizo*), followed by indigenous Amazonian (27.0%) and Afro Peruvian (12.3%). Most participants reported having an informal job (63.1%), while 33.6% reported being unemployed. Among the 71 unemployed participants, 27% were currently in school. There were no significant differences in HIV status between participants currently in school compared to those who were not currently in school. The majority of the study population (68.3%) reported earning under 1500 soles (US$ 410) per month. There were no significant differences in socio‐demographic characteristics by HIV status.

### 3.2 HIV and STIs

**Figure 1:**
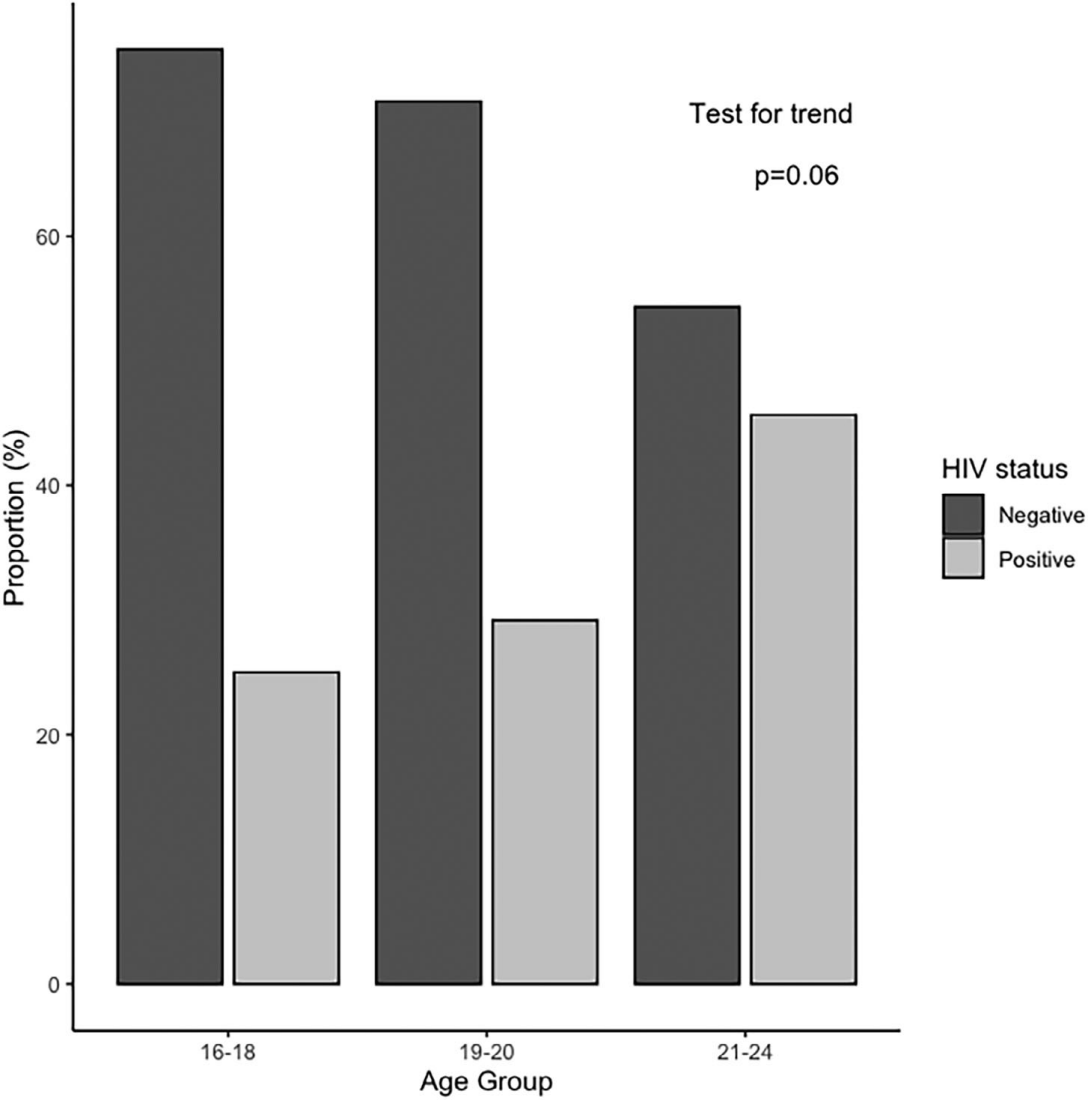
Proportion of participants testing positive and negative for HIV by age group.

Table 2 shows the prevalence of HIV and other STIs. HIV prevalence was 41.5% (95% CI: 33.9−49.4%). Of the 68 participants testing HIV positive, 79% (*n* = 54) self‐reported being HIV negative or not knowing their HIV status. As displayed in Figure 1, HIV prevalence increased by age, with the highest prevalence among 21‐ to 24‐year‐olds (45.7%) and lowest among 16‐ to 18‐year‐olds (25.0%) (*p* = 0.06 for trend). Over half of the participants (64.4%) tested positive for at least one STI. TPPA positivity for syphilis was 53.0% (95% CI: 45.1−60.8) and the prevalence of recent syphilis acquisition was 19.4% (95% CI: 12.7−28.4). The prevalence of chlamydia was 6.3% (95% CI: 3.1−11.1) and gonorrhoea was 12.3% (95% CI: 7.9−18.7). The prevalence of HIV co‐occurrence with another STI (syphilis, chlamydia, gonorrhoea, hepatitis B) was 39.1%, and 73.8% of participants had HIV or another STI.

**Table 2:** Prevalence of HIV and other STIs among young transgender women in Lima, Peru

	Count	Prevalence (%)
	*n*/*N*	(95% CI)
HIV	68/164	41.5 (33.9–49.4)
Syphilis (recent)^*^	21/108	19.4 (12.7–28.4)
Syphilis (All‐life exposure)^**^	87/164	53.0 (45.1–60.8)
Chlamydia	10/165	6.1 (3.1–11.1)
Gonorrhoea	20/162	12.3 (7.9–18.7)
Hepatitis B (anti core)	18/164	11.0 (6.8–17.0)
Hepatitis C	2/164	1.2 (0.2–5.8)
HIV and STI co‐occurrence^a^	52/163	31.9 (25.0–39.7)
≥1 STI^b^	105/163	64.4 (56.5–71.6)
HIV or STI^c^	121/164	73.8 (66.2–80.2)

^a^ HIV and at least other STI (syphilis, chlamydia, gonorrhoea, hepatitis B).

^b^ Syphilis (TPHA), chlamydia, gonorrhoea, hepatitis B.

^c^ Testing positive for at least one of HIV, syphilis (recent), chlamydia, gonorrhoea or hepatitis B.

* Determined by RPR titre >1:8.

** Determined by TPHA titre>1:80.

### 3.3 Sexual behaviour

Among the 192 participants with complete data on recent sexual partners, 154 (80.2%) reported cisgender male sexual partners, 12 (6.3%) reported cisgender female sexual partners, 6 (3.1%) reported transgender male sexual partners, 7 (3.6%) reported transgender female sexual partners and 10 (5.2%) reported sexual partners of another gender. Table 3 presents the sexual behaviours of participants, overall and by HIV status. In the past 6 months, 51.2% of participants reported engaging in receptive anal sex, 21.3% insertive anal sex and 6.6% insertive vaginal sex. While 20.4% of participants reported no recent sexual partners, 64.5% reported two or more recent sexual partners. Approximately half (47.9%) of participants reported engaging in condomless anal sex at least once in the past 6 months. While 145 participants (68.7%) reported a history of sex work, 107 (50.7%) reported engaging in sex work in the past 30 days and 29 (13.7%) reported accepting more money for not using condoms during sex work. There were no significant differences in reported sexual behaviours by HIV status.

**Table 3:** Recent sexual behaviour by HIV status among young transgender women in Lima, Peru

	All participants	Participants who completed HIV testing		
	Overall	HIV negative	HIV positive	
	*N* = 211	*N* = 96	*N* = 68	
	*n* (%)	*n* (%)	*n* (%)	*p*‐value^+^
Type of recent sexual activity (past 6 months)				
Engaged in receptive anal sex				0.61
Yes	108 (51.2%)	50 (52.1%)	36 (52.9%)	
No	65 (30.8%)	25 (26.0%)	21 (30.9%)	
Missing	38 (18.0%)	21 (21.9%)	11 (16.2%)	
Engaged in insertive anal sex				0.18
Yes	45 (21.3%)	18 (18.8%)	18 (26.5%)	
No	149 (70.6%)	70 (72.9%)	46 (67.6%)	
Missing	17 (8.1%)	8 (8.3%)	4 (5.9%)	
Engaged in insertive vaginal sex				0.46
Yes	14 (6.6%)	8 (8.3%)	5 (7.4%)	
No	180 (85.3%)	84 (87.5%)	55 (80.9%)	
Missing	17 (8.1%)	4 (4.2%)	8 (11.8%)	
Recent sexual partners (past 6 months)				
Number of recent sexual partners				0.75
None	43 (20.4%)	15 (15.6%)	14 (20.6%)	
One sexual partner	13 (6.2%)	5 (5.2%)	2 (2.9%)	
Multiple sexual partners	136 (64.5%)	67 (69.8%)	47 (69.1%)	
Missing	19 (9.0%)	9 (9.4%)	5 (7.4%)	
Recent condomless anal sex (past 6 months)				
Engaged in condomless anal sex (overall)				0.88
Yes	101 (47.9%)	50 (52.1%)	33 (48.5%)	
No	86 (40.8%)	33 (34.4%)	26 (38.2%)	
Missing	24 (11.4%)	13 (13.5%)	9 (13.2%)	
Engaged in condomless receptive anal sex				0.78
Yes	98 (46.4%)	49 (51.0%)	31 (45.6%)	
No	92 (43.6%)	35 (36.5%)	28 (41.2%)	
Missing	21 (10.0%)	12 (12.5%)	9 (13.2)	
Engaged in condomless insertive anal sex				0.42
Yes	23 (10.9%)	9 (9.4%)	11 (16.2%)	
No	171 (81.0%)	79 (82.3%)	52 (76.5%)	
Missing	17 (8.1%)	8 (8.3%)	5 (7.4%)	
Participation in sex work				
Ever engaged in sex work				0.13
Yes	145 (68.7%)	65 (67.7%)	50 (73.5%)	
No	64 (30.3%)	31 (32.3%)	16 (23.5%)	
Missing	2 (0.9%)	0 (0.0%)	2 (2.9%)	
Recently engaged in sex work (past 30 days)				0.31
Yes	107 (50.7%)	49 (51.0%)	37 (54.4%)	
No	100 (47.4%)	46 (47.9%)	28 (41.2%)	
Missing	4 (1.9%)	1 (1.0%)	3 (4.4%)	
Ever accepted more money for not using condoms				0.24
Yes	29 (13.7%)	12 (12.5%)	15 (22.1%)	
No	177 (83.9%)	82 (85.4%)	51 (75.0%)	
Missing	5 (2.4%)	2 (2.1%)	2 (2.9%)	

^+^ *p*‐values of the comparison of proportions between participants who tested HIV positive and HIV negative.

### 3.4 Psychosocial characteristics

Psychosocial characteristics of the sample are displayed in Table 4. The median age of self‐acknowledgement of gender identity was 13 (IQR: 8−16). Approximately half of the sample (52.7%) were not “out” about being trans in all aspects of their lives. Regarding medical gender affirmation, 49.8% reported ever using hormones, 20.9% ever injecting silicone and 15.2% having had at least one surgical procedure.

**Table 4:** Psychosocial characteristics by HIV status among young transgender women in Lima, Peru

	All participants	Participants who completed HIV testing		
	Overall *N* = 211	HIV negative *N* = 96	HIV positive *N* = 68	*p*‐value^+^
Gender affirmation				
Gender identity disclosure				0.31
“Out” in all areas of life (score = 35)	97 (47.3%)	40 (41.7%)	36 (52.9%)	
Not out in all areas of life (score < 35)	108 (52.7%)	52 (54.2%)	31 (45.6%)	
Missing	6 (2.8%)	4 (4.2%)	1 (1.5%)	
Age of self‐acknowledgement of gender identity (median [IQR])^a^	13.0 (8.0–16.0)	13.0 (10.0−16.0)	11.0 (7.5−15.0)	0.12
Ever injected silicone				0.25
Yes	44 (20.9%)	19 (19.8%)	18 (26.5%)	
No	165 (78.2%)	77 (80.2%)	49 (72.1%)	
Missing	2 (0.9%)	0 (0.0%)	1 (1.5%)	
Ever taken hormones				0.95
Yes	105 (49.8%)	49 (51.0%)	36 (52.9%)	
No	101 (47.9%)	45 (46.9%)	31 (45.6%)	
Missing	5 (2.4%)	2 (2.1%0	1 (1.5%)	
Ever had gender‐affirming surgery^b^				1.00
Yes	32 (15.2%)	13 (13.5%)	9 (13.2%)	
No	179 (84.8%)	83 (86.5%)	59 (86.8%)	
Missing	0 (0.0%)	0 (0.0%)	0 (0.0%)	
Experiences of violence				
Ever experienced violence				0.37
Yes	130 (61.6%)	63 (65.6%)	40 (58.8%)	
No	80 (37.9%)	33 (34.4%)	27 (39.7%)	
Missing	1 (0.5%)	0 (0%)	1 (1.5%)	
Experienced violence in past 3 months				0.67
Yes	45 (21.3%)	20 (20.8%)	14 (20.6%)	
No	165 (78.2%)	76 (79.2%)	53 (77.9%)	
Missing	1 (0.5%)	0 (0.0%)	1 (1.5%)	
Ever experience trans‐specific IPV				0.32
Yes	52 (24.6%)	20 (20.8%)	19 (27.9%)	
No	155 (73.5%)	75 (78.1%)	47 (69.1%)	
Missing	4 (1.9%)	1 (1.0%)	2 (2.9%)	
Experienced trans‐specific IPV in past 12 months				0.39
Yes	39 (18.5%)	15 (15.6%)	15 (22.1%)	
No	165 (78.2%)	78 (81.2%)	49 (72.1%)	
Missing	7 (3.3%)	3 (3.1%)	4 (5.9%)	
Mental health and substance use				
Recent psychological distress (past 30 days)				0.78
Moderate to severe psychological distress	42 (19.9%)	21 (21.9%)	12 (17.6%)	
Low to none psychological distress	165 (78.2%)	74 (77.1%)	55 (80.9%)	
Missing	4 (1.9%)	1 (1.0%)	1 (1.5%)	
Ever experienced traumatic event				0.70
Yes	60 (28.4%)	29 (30.2%)	20 (29.4%)	
No	150 (71.1%)	67 (69.8%)	47 (69.1%)	
Missing	1 (0.5%)	0 (0.0%)	1 (1.5%)	
PTSD symptoms (score ≥4/5)				0.48
Yes	43 (20.4%)	19 (19.8%)	15 (22.1%)	
No	167 (79.1%)	77 (80.2%)	52 (76.5%)	
Missing	1 (0.5%)	0 (0.0%)	1 (1.5%)	
Recent suicidal thoughts (past 6 months)				0.04
Yes	29 (13.8%)	18 (18.8%)	5 (7.4%)	
No	181 (85.8%)	78 (81.2%)	62 (91.2%)	
Missing	1 (0.5%)	0 (0.0%)	1 (1.5%)	
Ever attempted suicide				0.22
Yes	50 (23.7%)	29 (30.2%)	13 (19.1%)	
No	158 (74.9%)	66 (68.8%)	54 (79.4%)	
Missing	3 (1.4%)	1 (1.0%)	1 (1.5%)	
Recent suicide attempt (past 6 months)				0.02
Yes	21 (10.0%)	15 (15.6%)	3 (4.4%)	
No	189 (89.6%)	81 (84.4%)	64 (94.1%)	
Missing	1 (0.5%)	0 (0.0%)	1 (1.5%)	
Alcohol misuse				0.58
Yes (≥3 on AUDIT‐C)	135 (64.0%)	59 (61.5%)	47 (69.1%)	
No (<3 on AUDIT‐C)	23 (10.9%)	11 (11.5%)	7 (10.3%)	
Missing	53 (25.1%)	26 (27.1%)	14 (20.6%)	
Healthcare access				
Ever tested for HIV				0.66
Yes	127 (60.2%)	58 (60.4%)	41 (60.3%)	
No	82 (38.9%)	38 (39.6%)	26 (38.2%)	
Missing	2 (0.9%)	0 (0.0%)	1 (1.5%)	
Tested for STIs in past 6 months				0.60
Yes	54 (25.6%)	23 (24.0%)	18 (26.5%)	
No	143 (67.8%)	68 (70.8%)	44 (64.7%)	
Missing	14 (6.6%)	5 (5.2%)	6 (8.8%)	
Medical insurance				0.28
None	45 (21.3%)	29 (30.2%)	13 (19.1%)	
Private	64 (30.3%)	28 (29.2%)	18 (26.5%)	
SIS (public)	97 (46.0%)	36 (37.5%)	35 (51.5%)	
Missing	5 (2.4%)	3 (3.1%)	2 (2.9%)	
Heard of PrEP^c^				
Yes	46 (31.3%)	32 (34.0%)	14 (26.4%)	0.44
No	101 (68.7%)	62 (66.0%)	39 (73.6%)	
Missing	0 (0.0%)	0 (0.0%)	0 (0.0%)	
Past 30 days PrEP use^c^				
Yes	8 (5.4%)	6 (6.4%)	2 (3.8%)	0.71
No	139 (94.6%)	88 (93.6%)	51 (96.2%)	
Missing	0 (0.0%)	0 (0.0%)	0 (0.0%)	
Anticipated discrimination overall				
Overall score (median [IQR])	2.0 (2.0−3.0)	2.0 (1.9−3.0)	2.0 (2.0−3.0)	0.72
Anticipated healthcare discrimination				
Agree or strongly agree	67 (31.8%)	33 (34.4%)	19 (27.9%)	
Neither, disagree, strongly disagree	140 (66.4%)	60 (62.5%)	48 (70.6%)	
Missing	4 (1.9%)	3 (3.1%)	1 (1.5%)	

Abbreviations: IQR, interquartile range; PTSD, post‐traumatic stress disorder.

^a^ Missing *n* = 11 (5.2%).

^b^ Defined as breast implants, face/neck surgery or genital surgery.

^c^ Only among participants, self‐reporting HIV negative or HIV status unknown (*n* = 147).

^+^ *p*‐values of the comparison of proportions between participants who tested HIV positive and HIV negative.

### 3.5 Mental health and violence experiences

The majority of participants (61.6%) reported experiencing violence at some point in their lifetime (psychological, physical or sexual) and 21.3% reported experiencing violence in the past 3 months (Table 4). Transgender‐specific intimate partner violence was reported by 24.6% of participants, with 18.5% of participants reporting experiences in the past 3 months. Regarding current mental health, 19.9% endorsed moderate to severe psychological distress and 20.4% PTSD symptoms. Approximately one in four (23.7%) reported ever attempting suicide and 10.0% reported attempting suicide in the past 6 months. Alcohol misuse was found in 64.0% of participants (Table 4). The most common type of violence experienced over the lifetime was psychological violence (56.4%), followed by physical violence (44.5%) and sexual violence (26.1%).

For healthcare access (Table 4), most participants reported having medical insurance (46.0% public insurance, 30.3% private). Only 60.2% of participants reported ever participating in HIV testing, and only 25.6% reported an STI test in the past 6 months. The majority of participants (68.7%) had not heard of PrEP and only 5.4% had taken PrEP in the past month. One‐third (31.8%) anticipated experiencing discrimination in healthcare settings.

### 3.6 Multivariable analysis

Table 5 presents a multivariable Poisson regression analysis estimating the prevalence of HIV acquisition among participants, considering socio‐demographic and behavioural characteristics. Adjusted for age and education, participants who reported accepting more money for not using condoms during sex work were more likely to test positive for HIV acquisition (PR = 1.50, 95% CI: 1.01–2.23), while recent sex work alone was not significantly associated with HIV acquisition. Testing positive for an STI was significantly associated with testing positive for HIV acquisition, in both the model adjusted for age and education and in the fully adjusted multivariable models.

**Table 5:** Bivariate and multivariable models: factors associated with HIV acquisition among young transgender women in Lima, Peru^*^

	Bivariate analysis	Adjusted for socio‐demographics^a^	Multivariable model 1^b^	Multivariable model 2^c^
	HIV	HIV	HIV	HIV
	PR (95% CI); *p*‐value	aPR (95% CI); *p*‐value	aPR (95% CI); *p*‐value	aPR (95% CI); *p*‐value
Age (per 1 year increase)	1.08 (0.98−1.18); *p* = 0.12	–	1.04 (0.95−1.15); *p* = 0.39	1.05 (0.95−1.15); *p* = 0.37
Education		–		
< High school (ref)	1.00 (ref)		1.00 (ref)	1.00 (ref)
≥ High school	0.92 (0.62−1.37); *p* = 0.70		1.04 (0.70−1.55); *p* = 0.84	1.05 (0.71−1.56); *p* = 0.79
Ethnicity		–	–	
Mestiza (ref)	1.00 (ref)			
Afro‐Peruvian	0.62 (0.29−1.33); *p* = 0.22			
Indigenous	1.20 (0.80−1.80); *p* = 0.39			
White	0.43 (0.12−1.58); *p* = 0.21			
Don't know/other	1.13 (0.64−2.00); *p* = 0.67			
Indigenous (ref = no)	1.35 (0.95−1.93); *p* = 0.10	–	–	1.19 (0.82−1.71); *p* = 0.36
Recent suicidal thoughts (ref=none)	0.49 (0.22−1.09); *p* = 0.08	0.53 (0.24−1.19); *p* = 0.13	0.56 (0.25−1.25); *p* = 0.16	0.58 (0.25−1.31); *p* = 0.19
Recent sex work (ref=none)	1.14 (0.78−1.66); *p* = 0.51	1.11 (0.76−1.63); *p* = 0.59	–	–
Accepted money for not using condoms (ref=no)	1.45 (0.97−2.16); *p* = 0.07	1.50 (1.01−2.23); *p* = 0.05	1.45 (0.98−2.15); *p* = 0.07	1.41 (0.94−2.12); *p* = 0.09
Any STI (ref = none)	1.91 (1.19−3.08); *p* = 0.01	1.83 (1.12−2.97); *p* = 0.02	1.74 (1.06−2.85); *p* = 0.02	1.70 (1.03−2.79); *p* = 0.04

Abbreviations: aPR, adjusted prevalence ratios; PR, prevalence ratios.

^a^ Adjusted for age and education (recent suicidal thoughts: *N* = 162; recent sex work: *N* = 159; accepted money for not using condom: *N* = 159; any STI: *N* = 161).

^b^ Model 1: age, education, recent suicidal thoughts, accepted money for not using condoms, any STI (*N* = 158).

^c^ Model 2: age, education, recent suicidal thoughts, accepted money for not using condoms, any STI, Indigenous identity (*N* = 158).

* Participants missing data for one or more covariates were excluded from the analysis.

## 4 DISCUSSION

This study assessed the HIV epidemic among an extremely vulnerable group of YTW living in Lima, Peru. Laboratory‐confirmed HIV prevalence in our sample was 41.5%—of which 79% were newly identified cases—and STI co‐occurrence was 31.9%. HIV prevalence appears higher than what has been assessed in other studies in Peru, and other countries of the region such as Argentina, Uruguay, Brazil or Paraguay [1, 25, 26], though methodological differences prohibit direct comparability. The trend of increasing HIV prevalence across age found in this study, which may indicate cumulative effects of vulnerability, suggests the potential impact of earlier intervention.

Our results highlight that the HIV epidemic for YTW in Lima, Peru is situated in the context of widespread stigma and social exclusion, including economic vulnerabilities, violence victimization and the mental health sequelae of transphobic stigma that starts early in life.

YTW in this sample experienced constrained educational and economic opportunities. More than half of participants reported sex work in the last 30 days to support themselves economically. Lifetime and recent experiences of violence were common, including psychological, physical and sexual violence.

Although the population reached in this study is not representative of the whole YTW population, our findings parallel the conditions of marginalization and exclusion that we described in adult TW in Peru more than a decade ago [2]. The Peruvian legal framework does not recognize the gender identity of transgender people, which is an obstacle to accessing basic rights such as health, education or work [27]. Furthermore, other public policies protecting the rights of transgender people are almost non‐existent in the country [28].

This paper reports transphobic violence including transgender‐specific intimate partner violence, building on previous literature among Peruvian adult TW [29]. Previous studies have shown gender‐based discrimination to be positively associated with HIV acquisition among TW in Brazil [25], and several studies around the world have described a similar context of violence and marginalization [30, 31, 32].

More than one‐quarter of YTW sampled experienced a lifetime traumatic event, and current PTSD symptomatology was high. The mental health context surrounding HIV also included suicidality, with one in four participants reporting a suicide attempt in their lifetime, a high prevalence of current psychological distress and alcohol misuse. Findings underscore the urgent need to develop and assess comprehensive mental health support and programmes tailored to the specific needs of YTW, to address the complex interplay between mental health challenges, HIV vulnerability and overall wellbeing.

Approximately half of the sample reported condomless receptive anal sex conferring sexual HIV acquisition or transmission risk. HIV and STI testing uptake were suboptimal and situated alongside more than one‐third of the sample reporting anticipated discrimination in healthcare settings. Experiences of discrimination can prevent access to health services, including HIV prevention and care [8, 33]. Two‐thirds of the sample had never heard of PrEP and uptake was extremely low (4.7%).

Few statistically significant differences were found between YTW by HIV serostatus. Engaging in sex work itself was not associated with HIV positivity. However, accepting more money for condomless sex during sex work was associated with an HIV‐positive serostatus in an age‐ and education‐adjusted multivariable model. Sexual and economic exploitation, situated within widespread transmisogyny and violence, can render YTW at an extreme structural disadvantage resulting in the imperative to accept more money for condomless sex during sex work [5, 6, 7].

These findings indicate an immediate opportunity and need for early programme efforts to address the HIV epidemic among YTW in Peru. In 2016, the Peruvian Ministry of Health approved guidelines to provide integrated and gender‐affirming care for TW, as part of HIV care programmes [34]. Nonetheless, it has not yet been adopted by most health establishments, and in some facilities, it stopped after the COVID‐19 pandemic [35].

The new HIV prevention guidelines for key populations in Peru include the provision of PrEP out of cost in primary care facilities, which started in November 2023 [36]. Though the scale‐up of PrEP provision is promising, access to services among TW is a bottleneck to widespread coverage of interventions [37]. A previous PrEP implementation study conducted in Brazil, Mexico and Peru showed challenges with engagement and adherence to PrEP among TW, fostered by constrained access to services and mistrust of health institutions [38]. In our study, 39% of participants had never tested for HIV. Additionally, 79% of those who tested positive for HIV during the implementation self‐reported being HIV negative or not knowing their HIV status before participating.

Structural interventions tackling broader determinants of health, such as guaranteeing protective legal frameworks (e.g. gender identity laws), labour inclusion programmes or food assistance initiatives for people living in extreme poverty, have shown to be feasible and to potentiate access to health services among transgender people in countries of the region like Argentina or Uruguay [34]. Moreover, our results underscore the need to design and evaluate strategies that integrate health provision with other protection systems focused on youth, gender‐based violence and discrimination against gender minorities.

There are several limitations of this study. First, this was a convenience sample of YTW recruited through peer networks and may not be representative or generalizable to all YTW in Peru. Second, this was a cross‐sectional study so findings are associational only and causality cannot be inferred. Third, the median age of participants was 23 and few YTW ages 16−17 years were sampled; thus, findings may overrepresent those ages 18−24 years. However, the study demonstrates a substantial unmet need for biomedical HIV prevention and the ability to recruit a vulnerable youth sample underserved by current HIV prevention efforts and in need of public health initiatives. Future research is needed to reach younger age TW and girls, including methodological research on how to reach the youngest age groups, particularly given the high burden of HIV and STI co‐occurrence already seen in the current sample and the need for early intervention.

## 5 CONCLUSIONS

The HIV burden among Peruvian YTW sampled was alarmingly high. Findings show the multiple mental health and psychosocial vulnerabilities faced by YTW that can intersect to fuel HIV vulnerability and STI co‐morbidities in YTW and need to be further studied. Furthermore, these findings emphasize the necessity to design and evaluate programmes addressing the root systems driving HIV vulnerabilities in YTW. To protect rights and wellbeing, initiatives should strengthen education, employment, and combat sexual exploitation, violence and transphobia. Healthcare awareness must be heightened for unhindered access to HIV/STI testing, prevention and care. Effective programmes in Peru must be youth‐oriented, developmentally informed and gender‐affirming.

## COMPETING INTERESTS

The authors declare to have no competing interests to disclose.

## AUTHORS’ CONTRIBUTIONS

AS‐S, SRL and AP‐B conceptualized the study; LH and FL managed the implementation of the study. SLR supervised the laboratory component of the study. SRL, AP‐B, RA‐R, SLR and AS‐S oversaw the methodology and implementation. DA, SRL and AS‐S conducted the data analysis. AS‐S, SRL and AP‐B wrote the original draft; all co‐authors contributed and reviewed the final version.

## FUNDING

Research reported in this manuscript was supported by the National Institute of Mental Health of the National Institutes of Health under award number NIH R21MH118110 (“HIV risk and psychosocial health among transgender women in Peru”; MPI: Reisner & Silva‐Santisteban). The content is solely the responsibility of the authors and does not necessarily represent the official views of the National Institutes of Health. Dr. Perez‐Brumer's time was supported by the Canadian Institutes of Health Research Grant [CRC‐2021‐00132; Canada Research Chair, Tier 2 PI: Perez‐Brumer].

## Supporting information


**Supplemental Table 1**: Characteristics of Young Transgender Women Participants who Completed HIV/STI Testing.

## Data Availability

The data that support the findings of this study are available on request from the corresponding author. The data are not publicly available due to privacy or ethical restrictions.
