# Supplementary material for: HIV vulnerabilities and psychosocial health among young transgender women in Lima, Peru: results from a bio‐behavioural survey
Source: J Int AIDS Soc. 2024 Jul 23;27(7):e26299. doi: 10.1002/jia2.26299 (PMC11264345; doi:10.1002/jia2.26299)
Supplement: Supplementary file 1 — Supplemental Table 1: Characteristics of Young Transgender Women Participants who Completed HIV/STI Testing. [file JIA2-27-e26299-s001.docx]

# **Supplemental** **Table 1: Characteristics of Young Transgender Women Participants who Completed HIV/STI Testing**

|  | **Did not participate in HIV testing**  **N = 47**  **n (%)** | **Participated in HIV testing**  **N = 164**  **n (%)** | **P-value** |
| --- | --- | --- | --- |
| **Age in years (median [IQR])** | 22 (20.0-24.0) | 23 (21.0-24.0) | 0.29 |
| **Born in Lima/Callao - Yes** | 25 (53.2%) | 69 (42.1%) | 0.33 |
| **Highest level of education**  Elementary school or less  Secondary school, incomplete  Secondary school, complete  Post-secondary, incomplete  Post-secondary, complete  Missing | 3 (6.4%)  9 (19.1%)  15 (31.9%)  15 (31.9%)  5 (10.6%)  0 (0.0%) | 8 (4.9%)  38 (23.2%)  61 (37.2%)  43 (26.2%)  13 (7.9%)  1 (0.6%) | 0.88 |
| **Ancestry and customs**  Indigenous Andean  Indigenous Amazonian  Indigenous (other)  Afroperuvian  White  Mestiza  Other  Missing | 2 (4.3%)  13 (27.7%)  3 (6.4%)  3 (6.4%)  4 (8.5%)  17 (36.2%)  0 (0.0%)  5 (10.6%) | 5 (3.0%)  44 (26.8%)  7 (4.3%)  23 (14.0%)  11 (6.7%)  55 (33.5%)  3 (1.8%)  16 (9.8%) | 0.85 |
| **Employment status**  Full time  Part time  Informal  Unemployed  Missing | 3 (6.4%)  3 (6.4%)  24 (51.1%)  17 (36.2%)  0 (0.0%) | 12 (7.3%)  6 (3.7%)  88 (53.7%)  54 (32.9%)  4 (2.4%) | 0.74 |
| **Monthly household income**  <300 soles  300 – 500 soles  501 – 1500 soles  <1501 soles  Missing | 6 (12.8%)  12 (25.5%)  11 (24.4%)  8 (17.0%)  10 (21.3%) | 37 (22.6%)  39 (23.8%)  39 (23.8%)  23 (14.0%)  26 (15.9%) | 0.62 |
| **Multiple recent sexual partners (2+)** | 22 (46.8%) | 114 (69.5%) | 0.02 |
| **Engaged in condomless anal sex (overall) - Yes** | 18 (38.3%) | 83 (50.6%) | 0.02 |
| **Ever accepted more money for not using condoms - Yes** | 2 (4.3%) | 27 (16.5%) | 0.10 |
| **HIV and STI testing**  Ever tested for HIV  Tested for STIs in past 6 months | 28 (59.6%)  13 (27.7%) | 99 (60.4%)  41 (25.0%) | 0.64  0.93 |
| **Self-reported HIV status**  Negative  Positive  Don’t know  Refuse to answer | 27 (57.4%)  3 (6.4%)  14 (29.8%)  3 (6.4%) | 82 (50.0%)  16 (9.8%)  29 (23.8%)  27 (16.5%) | 0.26 |
